# Gut microbiome at the crossroad of genetic variants and behavior disorders

**DOI:** 10.1080/19490976.2023.2201156

**Published:** 2023-04-23

**Authors:** Lingsha Cheng, Haoqian Wu, Zhuo Chen, Haiping Hao, Xiao Zheng

**Affiliations:** a State Key Laboratory of Natural Medicines, School of Pharmacy, China Pharmaceutical University, Nanjing, Jiangsu, China; b Laboratory of Metabolic Regulation and Drug Target Discovery, School of Pharmacy, Jiangsu Province Key Laboratory of Drug Metabolism, China Pharmaceutical University, Nanjing, Jiangsu, China

**Keywords:** Gut dysbiosis, microbiome-gut-brain axis, neuropsychological disorder, genetic risk, microbial metabolites

## Abstract

Genetic variants are traditionally known to shape the susceptibility to neuropsychiatric disorders. An increasing number of studies indicate that remodeling of the gut microbiome by genetic variance serves as a versatile regulator of gut-brain crosstalk and behavior. Evidence also emerges that certain behavioral symptoms are specifically attributed to gut microbial remodeling and gut-to-brain signals, which necessitates rethinking of neuropsychiatric disease etiology and treatment from a systems perspective of reciprocal gene-microbe interactions. Here, we present an emerging picture of how gut microbes and host genetics interactively shape complex psychiatric phenotypes. We illustrate the growing understanding of how the gut microbiome is shaped by genetic changes and its connection to behavioral outcome. We also discuss working strategies and open questions in translating associative gene-microbiome-behavior findings into causal links and novel targets for neurobehavioral disorders. Dual targeting of the genetic and microbial factors may expand the space of drug discovery for neuropsychiatric diseases.

## Introduction

The incidence of neuropsychiatric diseases is ever on the increase in modern society. Cumulative genome-wide association study (GWAS) findings have highlighted association of common genetics variants with neuropsychiatric disorders typically including autism spectrum disorder (ASD)^1^, major depressive disorder (MDD)^2^, bipolar disorder (BD), and schizophrenia^3^ (Table 1). Although the contribution of heritability varies, deciphering their links to behavioral symptoms provides a promising route to therapeutic innovation and clinical care. A traditional view is that, by directly impacting brain development and function, genetic variants are the primary drivers of the behavioral symptoms. Indeed, the genetic-risk loci are increasingly linked to parameters of neuroinflammation, neurogenesis, synaptic, or neurocircuit function, which are commonly viewed as fundamental to disease phenotypes^17,18^. However, given the inherent complex etiology of neuropsychiatric diseases, a major unanswered question is how the genetic variance may interact with other environmental factors underlying the complex behavioral phenotypes.

**Table 1.** Representative risk genes of neurobehavioral disorders and gut microbial changes reported in these patients.

Neurobehavioral Disorders	Candidate risk genes	Gut microbial changes		Refs
		Positive association/Higher abundance in patients	Negative association/Lower abundance in patients	
Autism spectrum disorder	*Negr1;Ptbp2;Cadps;Fezf2;Tmem33;Dcaf4l1;Slc30a9;Bend4;Nudt12;Kcnn2;Mms22l;Pou3f2;Kmt2e;Chd8;Srpk2;C8orf74;Sox7;Shank3;Pinx1;Mroh5;Mark3;Ckb; Trmt61a;Bag5;Apopt1;Klc1;Cntnap2;Xrcc3;Macrod2; Xrn2; Kiz; Nkx2–4; Nkx2–2*	family *Prevotellaceae;* genus *Bacteroides;* genus *Clostridium*; genus *Faecalibacterium;* genus *Phascolarctobacterium;* genus *Collinsella;* genus *Corynebacterium;* genus *Dorea;* genus *Lactobacillus*	phylum *Bacteroidetes;* family *Acidaminococcaceae*; genus *Lachnoclostridium*, genus *Tyzzerella subgroup 4;* genus *Flavonifractor*; genus *Coprococcus*; genus *Bifidobacterium*; genus *Alistipes*; genus *Bilophila*; genus *Dialister*; genus *Veillonella*	^4–7^
Major depressive disorder	*Ccdc71; Acbrl1; Rp11-3b7.1; Tlr4; Celf4; Fcf1; Fads1; Arel1; Fads2; Ephx2; Chrna7; Ylpm1; Sppl3; Tiaf1; Traf3; Ccdc36; Sorcs3; Klhdc8b; Tmem258; Lamb2; Nlrp3*	phylum *Actinobacteria*; class *Bacilli;* order *Enterobacterales;* family *Enterobacteriaceae;* family *Bifidobacteriaceae;* family *Coriobactericaceae;* family *Lachnospiraceae;* family *Streptococcaceae;* genus *Eggerthella;* genus *Olsenella;* *genus Collinsella;* *genus Lactobacillus*; genus *Oscillibacter*; genus *Blautia;* genus *Erysipelotrichaceae incertaesedis;* genus *Holdemania*; genus *Streptococcus;* genus *Desulfovibrio;* genus *Paraprevotella*	phylum *Bacteroidetes;* family *Prevotellaceae;* genus *Dialister*; genus *Coprococcus*; genus *Sutterella;* *genus Faecalibacterium;* *genus Clostridium cluster XIVa;* *genus Coprococcus*	^8–10^
Bipolar disorder	*Tmem108; Rhebl1; Nek4; Glt8b1; Mcm3ap; Cacna1; Ank3; Ncan; Scn2a; Pou3f2; Stk4; Add3; Furin; Hapln4; Ac092661.1; Dclk3; Tmem258; Rp5-882c2.2; Gnl3; Lman2l; Pacs1; Lrrc57; Pacsin2; Htr6; Mchr1*	Class *Betaproteobacteria* *genus Bifidobacterium;* *genus Oscillibacter;* genus *Megasphaera;* genus *Enterococcus*; genus *Flavonifractor*; genus *Streptococcus*	genus *Faecalibacterium;* genus *Roseburia;* genus *Ruminococcus*	^5,11–13^
Schizophrenia	*Grin2a; Sp4; Stag1; Fam120a; Bcl11b; Cacna1c; Rere; Foxp1; Myt1l; Slc39a8*	phylum *Firmicutes;* class *Actinobacteria;* order *Enterobacteriales;* family *Enterobacteriaceae;* *genus Haemophilus;* *genus Lachnoclostridium;* *genus Prevotella;* *genus Escherichia;* *genus Shigella;* *genus Veillonella;* *genus Megasphaera*	genus *Bifidobacterium*; genus *Coprococcus*; genus *Ruminococcaceae*; genus *Bacteroides*; genus *Haemophilus;* genus *Streptococcus;* genus *Roseburia*	^5,10,13,14^
Rett syndrome	*Mecp2*	family *Bacteroidaceae*; family *Erysipelotrichaceae;* genus *Escherichia*; genus *Clostridium*; genus *Sutterella*	family *Ruminococcaceae*	^15,16^

The gut microbiome can modulate host behaviors in a very powerful manner via the gut-brain axis^19,20^. Interestingly, gut dysbiosis has been observed as a common trait for neuropsychiatric disorders, and emerging data are suggesting that host genetics and the gut microbiome interdependently regulate different complex behaviors in genetic neurological disorders^21^. Therefore, it is reasonable to propose that dissecting the mechanisms through which host and microbial factors regulate complex behaviors will not only expand our understanding of neurobehavioral disorders but may also broaden the way of novel therapies. Here, we present a growing picture from animal studies that gut microbe interaction with host genetics to interactively shape complex psychiatric phenotypes. We discuss the challenges and potential strategies in gaining mechanistic insights into the genetic-microbe interplay in behavior control, with a major focus on microbial metabolites as signaling molecules for drug discovery. We also envision how advances in this frontier may potentially change the way we view and treat these complex mental illnesses in the clinic.

## Gut dysfunction and dysbiosis in neuropsychiatric diseases

In addition to brain-centric symptoms, somatic complications have emerged as a common clinical problem for neuropsychiatric diseases. Gut dysfunction, among others, seems to be the most common comorbidity with the behavioral changes^22^. For example, a spectrum of gastrointestinal disorders, such as constipation, diarrhea, abdominal pain, vomiting, and poor nutritional absorption, have been reported in up to 90% of children with ASD^23^. Likely, MDD patients largely exhibit abnormality in gut motility, dyspepsia, and increased susceptibility to colitis. Also, patients with BD exhibit an increased frequency of gastrointestinal illnesses, such as inflammatory bowel disease, which has been mechanistically connected to microbial community function^24^. Gut dysfunction and associated metabolic and immune changes could in turn aggravate the behavioral symptoms, such as anxiety and social disruption^25^, thus forming a vicious cycle that calls for multi-dimensional intervention.

Changes in gut physiology are well known to affect the colonization and fitness of the commensal microbial community. In addition, altered feeding behavior and nutritional status, commonly observed in neuropsychiatric diseases, are powerful in shaping the gut microbial configuration^26^. Not surprisingly, gut microbial remodeling has now been extensively observed in patients with neuropsychiatric disorders (Table 1). For example, there is early metagenomic and metabolic evidence suggesting that ASD is accompanied by gut dysbiosis^27^, and a recent large-cohort study confirmed progressive deviation in the development of gut microbiota in children with ASD^28^. Intriguingly, ASD risk genes are directly involved in regulating gut nutrition processing. For example, human patients with *Shank3* mutation-associated autism show lower expression of zinc transporter in enterocytes^29^. *Chd8* mutation, a validated risk factor for ASD, is associated with slower intestinal motility and discomfort in humans, which is also proved in mouse and zebrafish models^30^. Rett syndrome (RTT), a progressive neurological disorder characterized by autism-like behaviors, is thought to be caused by mutations in the *Mecp2* gene^31^. Recent study has moved the field one step further by showing that RTT patients harbored a gut microbiome featuring reduced microbial richness and a dominance of *Bifidobacterium*, *Actinomyces*, *Lactobacillus* and *Enterococcus*^32^. In addition, ample clinical findings show that the gut microbiome is altered in MDD patients^8,33^.

**Figure 1.**
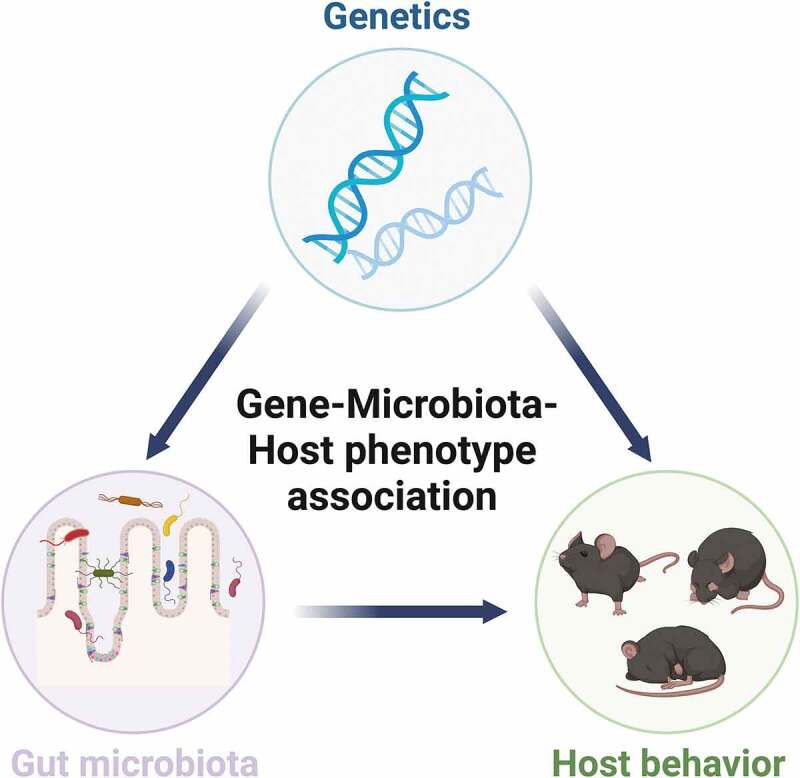
A triangular connection exists among host genetic variants, gut microbiome and neurobehavioral phenotype.

Consistently, genetic manipulations in animals induce both behavioral changes and gut microbiome remodeling (Table 2). For example, a study in *Chd8*± mice revealed changes in gut microbial structure and amino acid transporters, which were linked to an increased glutamate/γ-aminobutyric acid (GABA) ratio in the brain^34^. Similarly, a previous study in *Shank3*^KO^ mice reported a significantly different gastrointestinal morphology and altered microbial composition^41^. A recent study in RTT mice also reported alteration of gut microbiome across postnatal development as behavioral symptoms appear and progress^42^. Together, the extensive observation of gut microbial remodeling in clinical patients and preclinical models sheds an additional layer of insights into neuropsychiatric diseases and genetic underpinnings (Figure 1).

**Table 2.** Preclinical studies exploring gut microbial alteration and its role in genetic-associated behavioral changes.

Genetic variant	Behavioral phenotype	Microbial changes	Functional validation method	Role of microbial remodeling	Refs
*Chd8*^+/−^	ASD-like anxiety, impaired social interaction, learning and memory deficits	*Bacteroides uniformis ↓*	Co-housing, mono-colonization	Increased excitory/Inhibitory (E/I) ratio	^34^
*Shank3B*^−/−^	ASD-like social deficits	*Lactobacillus reuteri ↓*	Mono-colonization	Increased level of oxytocin	^35^
*Shank3*^−/−^	ASD-like social deficits, repetitive behaviors	*Lactobacillus reuteri ↓*	Mono-colonization	GABA receptor subunits, oxytocin signaling	^36^
*Cntnap2*^−/−^	ASD-like social deficits	*Lactobacillus reuteri ↓*	Co-housing, fecal microbiota transplantation，mono-colonization	Increased tetrahydrobiopterin (BH4) metabolism pathway	^21^
*EphB6*^−/−^	Stereotyped behavior and social deficits, accompanied by anxiety-like behavior	*Mucispirillum ↓*	Fecal microbiota transplantation	Vitamin B6 homeostasis, decrease in dopamine	^37^
*Nlrp3*^−/−^	Depression and anxiety-like behavior	uncharacterized	Fecal microbiota transplantation	Astrocyte dysfunction, expression of circHIPK2	^38^
*Ephx2*^−/−^	Depressive-resilient phenotypes	*Faecalibaculum rodentium ↓*	Fecal microbiota transplantation, mono-colonization	Systemic inflammation, and synaptic proteins in the prefrontal cortex	^39^
*Chrna7*^−/−^	Depression-like behavior	uncharacterized	Fecal microbiota transplantation	synaptic proteins in the prefrontal cortex	^40^

## Gut microbiome contributes to genetic variance associated behavioral changes

A link between the brain and gut microbiome has long been surmised^43^. In 2009, pioneering studies implied that the altered gut microbiota may be correlated with social or learning behavior changes in rodents^44,45^. In the following several years, causal evidence for the role of gut microbiome in host nervous system development and behavior continued to accumulate^46–48^. To date, the gut microbiome has been firmly established as a critical regulator of gut-brain communication and behavior^49^. In line with the notion that neuropsychiatric disorders involve a complex interaction of gene-environmental factors, here we provide recent evidence from animal studies showing that gut microbial remodeling is causally linked to behavioral changes induced by genetic variance.

### ASD

ASD is traditionally thought to be a neurological disease with a high heritability. Studies in 2013 have shown direct evidence that gut microbiota could modulate autism-related behavior^47^. A more recent study transplanting gut microbiota from human donors with ASD to germ-free (GF) mice showed that ASD microbiota was sufficient to induce hallmark autistic behaviors, accompanied by discrete metabolome profiles^50^. Intriguingly, treatment of the BTBR ASD mouse model with key differential microbial metabolites improved behavioral abnormalities and modulated neuronal excitability–inhibition balance in the brain, providing further causal evidence for microbial factors. Interestingly, treatment with *Lactobacillus reuteri* (*L. reuteri*) was shown to generally rescue social deficits in *Shank3b*^KO^ and other environmental and idiopathic ASD models^35^. Also, of interest is the finding that *L. reuteri6475* intervention selectively rescued the social deficits but not the hyperactivity phenotype in *Cntnap2*^KO^ mice by bolstering the oxytocinergic system^21^. Together, these studies provide strong evidence that the gut ecosystem plays an important regulatory role in many aspects of ASD symptomatology.

### MDD

MDD is a polygenic disease, and a most recent GWAS covering over 1.2 million participants identified 178 genetic risk loci and 223 independently significant single-nucleotide polymorphisms (SNPs) that were associated with the disorder at genome-wide significance^2^. However, the heritability estimates for MDD (40%) are lower than those for other neuropsychiatric illnesses (between 75% and 80%), implying that additional factors are involved. Several preclinical studies have shown that genetic manipulation can effectively modulate depression-like behavior, with the mechanism being partially attributed to the gut microbiota. For example, a recent study showed that the relative abundance of *Firmicutes*, *Proteobacteria*, and *Bacteroidetes* in the gut microbiota of *Nlrp3*^KO^ and WT mice was substantially different^38^. Notably, fecal microbiome transplantation (FMT) from the *Nlrp3*^KO^ mice alleviated stress-induced depressive-like behaviors in recipient mice. *Chrna7*^KO^ mice, which have a deficiency in α7 subtype of nicotinic acetylcholine receptors (α7 nAChR), exhibited depression-like behavior and gut microbial changes (increased *Lactobacillus animalis*, *Helicobacter ganmani*, and decreased *Muribaculum intestinale*). In a following study, FMT proved successful to transmit the depressive behavior to recipient mice via vagal nerve signaling^40^.

### BD and schizophrenia

GWAS reveals a genetic connection between schizophrenia and BD, and many of the risk genes have been linked to immunological response and inflammation^51^. Ample data point to poor-diversity and dysbiosis with respect to the abundance of *Faecalibacterium* and *Bacteroides* as possible traits and state-dependent characteristics of BD. Reduced butyrate production and richness also drives inflammation, which may be a previously unappreciated component of the pathophysiology driving BD^52^. Association also exists between gut microbial dysbiosis and the hypothalamic-pituitary-adrenal (HPA) axis dysregulation in BD, as a significant negative correlation between the count of *Bifidobacterium* and cortisol levels was recently found^53^. Notably, HPA axis dysfunction in BD may worsen intestinal permeability and gut microbial dysbiosis in turn. Supporting the role of gut dysbiosis in schizophrenia patients, colonization of *Streptococcus vestibularis*, a bacterium enriched in treatment naïve schizophrenia patients, causes social behavior abnormalities, and alters neurotransmitter levels in peripheral tissues in antibiotics-treated recipient mice^54^.

### Fragile X syndrome (FXS)

FXS is an inheritable neurodevelopmental disease characterized by autistic traits, such as mental retardation and impaired social communication or interaction. Recently, there is accumulating evidence that FXS caused by *Fmr1* deficiency is likely to be caused by alterations in gut microbiota. More recently, gut microbiome changes involving *Akkermansia*, *Allobaculum*, *Bifidobacterium*, *Odoribacter*, *Flexispira*, *Bacteroides*, and *Oscillospira* have been profiled in *Fmr1*^KO^ mice, and, of note, FMT from normal mice proved effective to mitigate autistic-like behaviors^55,56^.

### Amyotrophic lateral sclerosis (ALS)

ALS is a fatal neurodegenerative disease that causes progressive motor neuron loss. ALS patients often show behavioral symptoms such as depression and anxiety^57^. ALS is thought to have a substantial genetic component with a high heritability, and many of the gene variations (e.g., *Sod1, Tardbp, Fus, C9orf72*) that cause or predispose an individual to ALS have been identified^58^. In 2017, gut dysbiosis with a reduced population of butyrate-producing bacteria was reported in *Sod1* (*G93A*) mice, which was rescued by butyrate treatment^59^. Indeed, novel evidence indicating a disease-modifying role for the gut microbiome has recently emerged, with certain gut microbial strains being causally linked to the behavioral symptoms in *Sod1* and *C9orf72* model mice^60,61^. Therefore, it is reasonable to dissect neuropsychiatric disorders from the perspective of reciprocal host gene-microbiome interactions.

## Strategies for identifying psychoactive microbes

**Figure 2.**
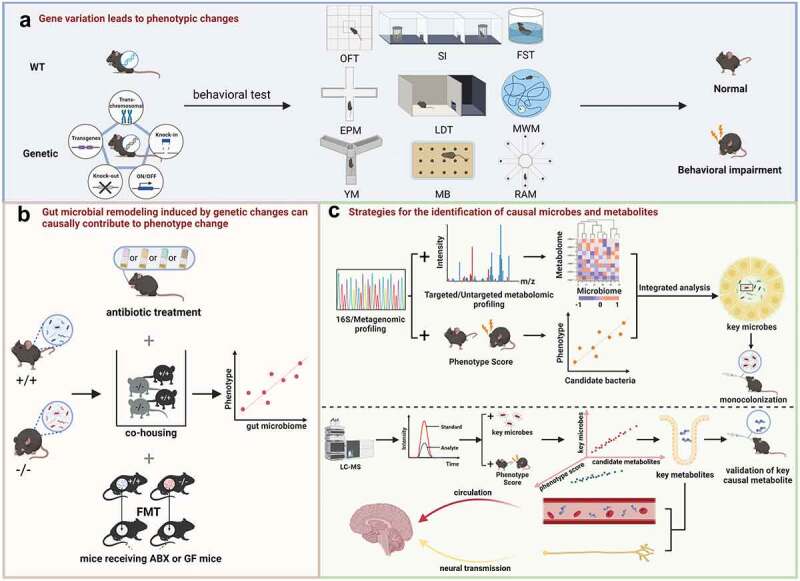
A tentative framework to disentangle gene-microbiome-phenotype interaction in complex neurobehavioral disease network. (a) Assessment of behavioral phenotypic changes after genetic manipulations. The commonly used behavioral tests used were open field test (OFT), social interaction (SI), forced swimming test (FST), elevated plus maze (EPM), light/dark test (LDT), Morris water maze (MWM), Y-maze (YM), marble-burying test (MB) and radial arm maze (RAM). (b) Causal role validation of gut microbial remodeling induced by genetic changes. The causal link between microbiome and behavioral phenotypes can be verified by antibiotics (ABX) treatment, co-housing, fecal microbiota transplantation (FMT) experiment with germ free (GF) mice. (c) Integrative strategies for the identification of causal microbes and metabolites. Combined analysis of microbiome and metabolomics, and a microbe-phenotype strategy to refine the catalog of differentially abundant microbes to most probably causal members followed by mono-colonization validation. Candidate metabolites identified by metabolomics are then assayed for the impacts on behavioral changes.

Microbe-wide association analysis, including 16S rRNA/18S rRNA/ITS and metagenomic sequencing, produce long lists of differential microbes. Due to the inherent complexity, diversity, and flexibility of the microbiome, standard correlative microbiome study is largely insufficient to elucidate the causal microbes. In further consideration of the complex routes of gut-brain communication^20^, unbiased identification of causal microbes in shaping neuropsychological diseases due to genetic variation is undoubtedly challenging. Here, we describe ongoing efforts and strategies seeking to answer this basic open question in microbiome study (Figure 2)，which may serve as a framework for further mechanistic studies in neuropsychiatric disease.

### Microbe-phenotype triangulation

Early pioneering studies by Gordon *et al*. describe the transplantation of combinatorial bacterial communities from human donors to gnotobiotic mice followed by mono-colonization validation^62^. This strategy features random fractionation of bacterial communities into subsets that are gavaged to recipient mice to identify the strains whose presence or absence best explain the observed phenotypic variation in metabolic and immune responses. Notably, by showing metabolite-microbe interactions under complex logics, this approach offers initial clues for deciphering the single and collective impact of different strains on the host metabolic phenotype. Therefore, this approach should facilitate mechanistic studies of how bacterial strains influence host behavioral phenotypes through metabolic and immune signals. A seminal work in recent years is the proposal of a microbe-phenotype triangulation strategy by Surana *et al*., which nicely refines the catalog of differentially abundant microbes to most probably causal members^63^. This strategy is based on the premise that co-housing mice with different microbial background can allow their flora to be recombined to form the ‘intermediate microbiome’ that blends the characteristics of the original flora. If the microbial effect on disease was dominant, mapping of microbe-phenotype relationships in parental and hybrid-microbiota mice would efficiently narrow down of the inherent noise in sequencing data and sort out key candidates. Of interest, this microbe-phenotype triangulation strategy is observed in the study of Regen *et al*., which proved useful to elucidate the microbial basis of the central nervous system autoimmunity shaped by *interleukin17* (*Il17*) genetic variant^64^. When healthy control and patients are carefully selected, such a microbe-phenotype triangulation strategy can be more generally applicable to human microbiome studies. However, this strategy has several caveats that may compromise the scalability. As a major concern, co-housing does not always result in intermediate phenotypes, and, instead, mono-directional transmission of one dominant phenotype could be observed^65^. Also, for rare species that may mediate a host phenotype, causal bacteria may be masked due to their relative low abundance^66^.

In essence, the microbe-phenotype triangulation strategy relies on the spatial transmission of causal microbes to enable comprehensive microbial analyses from microbially related mice^63^. For studies where co-housing is impractical, an alternative strategy would be dirty cage sharing, as recently shown by Guo *et al*.^67^. Specifically, in the search for key protective microbe in elite-survivors which were old male mice, a traditional cohousing approach with young male recipients might lead to fighting and injury to the older mice. By analyzing bacterial 16S rRNA genes in feces from donors and recipients after sharing dirty cages, it was shown that dirty cage sharing was effective in exchanging gut microbiota from donors to recipients.

### Select antibiotic disentanglement

Antibiotic cocktail comprising ampicillin, vancomycin, neomycin, and metronidazole is commonly used for assessing the role of gut microbiota in the host phenotype, although each antibiotic modulated the microbiota composition in a distinct way. Recent studies have also shown that the use of single antibiotics could also provide useful clues for identifying causal microbes. As an illustrative example, Miyauchi *et al*. showed that both depletion of the gut microbiota by antibiotic cocktail and oral treatment with ampicillin alone limited the development of experimental allergy encephalomyelitis (EAE)^68^. In consistence, ampicillin-treated mice showed a unique microbiota structure in the small intestine, and a novel strain named operational taxonomic unit (OTU)0002 was the sole sequence that was almost completely depleted only from the small intestine of ampicillin-treated mice. Notably, mono-colonization with OTU0002 resulted in an increased severity of EAE symptoms and frequency of T helper 17 (Th17) cells, both in the small-intestinal lamina propria and spleen. In another study seeking to identify gut bacterial species that affect social activity^69^, mice were treated with different combinations of antibiotics, which showed that a microorganism exclusively sensitive to neomycin appeared to be responsible for modulating social activity and corticosterone levels after social stress. Further study identified *Enterococcus faecalis* (ATCC 19,433) that promoted social activity and reduced activation of the HPA axis.

### Spatiotemporal microbial mapping

Gut microbial structure shows inherent plasticity and dynamics. To strengthen the confidence of causal microbe identification, temporal patterns of the gut microbiota could be mapped for integration with spatial profiling. In the study by Blacher *et al*., for example, several important time points were selected for bacterial sequencing along with the disease progression, and then the results were further integrated with spatial transmission features^60^. Eleven distinct commensal bacteria that would increase or decrease within the disease process were subsequently identified, and the role of each strain was verified by single bacterial colonization into antibiotic pre-treated model group. This strategy is of special value for causal microbe identification from human microbiome samples, which is complicated with high interindividual variance^70^. For example, in the search for irritable bowel syndrome (IBS) subtype-specific microbial composition and metabolites, longitudinal sampling effectively limited heterogeneity seen in cross-sectional microbiome studies^71^. The results showed that stool microbiota composition exhibited greater variability over time in patients with constipation-type IBS (IBS-C) compared to diarrhea-type (IBS-D) cohorts. In another clinical study involving hematopoietic cell transplantation patients, daily changes in circulating neutrophil, lymphocyte, and monocyte counts and more than 10,000 longitudinal microbiota samples were profiled to reveal consistent associations between gut bacteria and immune cell dynamics overtime^72^. These strategies are readily applicable to screen causal microbes underlying behavioral disorders.

### Computational algorithm and machine learning

Computational algorithm is also finding a wide application on identifying causal microbes or biomarkers from clinical large-scale databases of microbial community. Batch inconsistence of multi-population metagenomic data presents a challenge in identifying robust microbial markers. Addressing this issue, recently an algorithm called Network Module Structure Shift (NetMoss) has been devised, which proved useful to identify shared and unique microbial biomarkers from a network perspective^73^. This method is expected to enable reliable meta-analysis of metagenomic dataset from neuropsychiatry disease cohorts. Also, of note is the recent report of a machine learning-based framework that could jointly analyze paired host transcriptomic and gut microbiome data for deciphering host gene and gut microbiome interactions in disease^74^. This computational method can be applied to neuropsychiatric diseases for the identification of host gene-microbiome associations and key microbes that may influence behavioral outcomes.

It should be noted that current microbial mapping techniques have limitations in providing high-resolution and functional information, especially given the difficulties associated with assigning gene-based labels and gene-function correlation. The commonly used 16S rRNA sequencing technique reveals the bacterial genes they carry and the compounds they produce or consume. Thus, a better approach might be identifying not just the taxonomy, but the metagenomic signature, a matrix describing which genes are stored in which bacteria, and what is their abundance. This fact suggests that the screening and validation of potential causal microbes should consider strain and host origin differences and the sequencing depth. Moreover, even in the same model, intestinal site, and mucosal region-dependent changes of gut microbial composition should receive due attention in sampling and data interpretation^75^.

## Deciphering gut microbial signals in behavioral control

Current research in neurogenetics mostly seeks to answer how genetic variants regulate neuronal activity and circuit function in key brain regions using sophisticated cell-specific imaging, electrophysiology, and genetic manipulation techniques. Understanding how gut microbes communicate with the brain therefore holds another key to therapeutic target discovery. The past decade has witnessed huge advances in decoding the signaling mechanisms along the microbiota-gut-brain axis, which provide snapshots into the network connecting microbiome, brain and behavior including anxiety, cognition, nociception, and social interaction^20,76^. Concerning the neural transmission pathway, the message could be transmitted via the vagal or the sympathetic nervous system^77,78^. The effects of *L. reuteri* on social behavior were no longer present in vagotomized animals in a *Shank3B*^KO^ mouse model of autism^35^. Also, via direct synapsing with vagal nodose neurons, enteroendocrine cells (EEC) have emerged as a new sensory mechanism for rapid transmission of gut signals^79^. More recently, in zebrafish, microbial, pharmacological, or optogenetic activation of Trpa1+ EECs proved successful to directly stimulate vagal sensory ganglia and activate cholinergic enteric neurons by secreting the neurotransmitter 5-hydroxytryptamine (5-HT)^80^. Microbial signals can also transmit through the sympathetic nerve. A gut-brain-gut neural circuit, which regulates efferent sympathetic tone and gastrointestinal transit has been recently identified^78^. This effect was mainly attributed to microbial metabolites, such as short-chain fatty acids and bile acids, as well as microorganism-modulated GLP-1 release.

Bidirectional gut-brain signaling can also occur via circulating hormones, metabolites, cytokines, and other neuromodulatory molecules^69,75^, which appear to affect the brain in a direct or indirect manner. Elevated level of a microbial metabolite 4-ethylphenyl sulfate was previously reported in mice with social impairment^47^. A follow-up study showed that this gut-derived metabolite can enter the brain to affect oligodendrocyte function and neuronal myelin sheath pattern and promotes anxiety-like behavior in mice^81^. More recently, it is also reported that direct sensing of bacterial cell wall components (peptidoglycan) by hypothalamic Nod2+ neurons regulate appetite and body temperature in mice^82^. However, for those messengers that could not directly enter the brain, much more work is needed to fully address the exact mechanisms by which gut microbiota could distally impact on the brain and behavior. One common route is the sensing and transmission by neural pathway. For example, *Lactobacillus brevis* Bb14 (ATCC14869)-derived trehalose from sugar metabolism by the bacterial enzyme xylose isomerase activates peripheral octopaminergic neurons to regulate locomotor behavior in the fruit fly *Drosophila melanogaster*, while germ-free status or antibiotic treatment results in hyperactive locomotor behavior^83^. Recently, an inter-organ neural circuit for appetite suppression has been mapped, which conveys gut local GLP-1 signal to induce satiety^84^. Since gut microbes are intimately involved in the regulation of GLP-1 release, and GLP-1 has behavioral effects more than appetite control^85^, it would be of interest to further explore the involvement of this pathway in gut microbial regulation of behavior.

The involvement of immunologic signal pathways in microbiota-brain interaction has also been reported. For example, gut microbiome members such as *segmented filamentous bacteria* (SFB), *Bifidobacterium adolescentis*, and *Odoribacter splanchnicus* are well known to induce IL-17 production. Notably, SFB-induced susceptibility to stress-induced depressive-like behaviors in mice were causally linked to the expansion of Th17 cells, which accumulated in the hippocampus^86^. Context-dependent (during embryonic brain development or in the adult brain) effects of IL-17 in social behavior has been previously reported^87–89^, and, more recently, mucosa-associated fungi was shown to promote social behavior in mice via sensory neuron IL-17 receptor^86^.

Generally, the integration of ‘omics’ datasets, such as transcriptomics, metagenomics, proteomics, and metabolomics can be useful to pinpoint the regulatory microbes and signaling molecules underpinning host behavioral phenotype. This strategy is nicely depicted by the study of Sharon *et al*., which transplanted gut microbiota from human ASD donors into GF mice for mechanistic interrogation^50^. Through the inter-correlative analysis of species, metabolites, and behaviors of mice harboring human microbiota, candidate microbes, and metabolite that may mediate ASD behaviors were picked out for further validation. This strategy is also observed in profiling microbiota-host interaction in a *Chd8*± mouse model with ASD-like behavior, in which increased expression of amino acid transporters in the intestines of ASD mouse and high level of serum glutamine offer clues to understand the increased excitation/inhibition ratio in the brain^34^.

Given the inherent noise in the omics data, ‘trial and error’ screening is necessary, and several more criteria are worthy of consideration. This is elegantly demonstrated by the study of Blacher *et al*. to identify causal microbial metabolites in ALS from nicotinamide (NAM) and phenol sulfate, which had the highest metagenomic probability. By showing that phenol sulfate administration to mice did not relieve the symptoms of ALS mice, they focused on NAM for the following reasons: the marked differences in the metagenomic NAM biosynthetic pathway; the enrichment of NAM biosynthetic intermediates in serum upon supplementation with *Akkermansia muciniphila* (ATCC BAA-835); reduced abundance of genes from the gut-microbiome-derived tryptophan metabolizing pathway, which may be involved in NAM production; and the alteration of metabolites in the tryptophan pathway upon treatment with antibiotics or upon *Akkermansia muciniphila* supplementation^60^. Functional validation with NAM supplementation finally identified NAM as a key protective metabolite in ALS.

## Mechanisms underlying host genetics-induced gut microbial remodeling

Although host genetic variation is known to shape the gut microbial ecology^90,91^, there is still limited insights into how the composition and function of gut microbial community are altered. In essence, this reflects the interaction between the host’s mutated genes and microbial genes that shape their fitness and survival in the gut, as shown by recent studies in mice. A balanced gut microbial configuration is characterized by the dominance of obligate anaerobic members of the phyla *Firmicutes* and *Bacteroidetes*, which prevents dysbiotic expansion of facultative anaerobic microbes, such as *Enterobacteriaceae* in part by limiting the generation of host-derived nitrate and oxygen. Epithelial mitochondrial bioenergetics and metabolism has therefore proven to be a canonical pathway by which host genetic signals remodel the gut microbial configuration. A typical sensor/mediator is epithelial peroxisome proliferator-activated receptor γ (PPAR-γ), which drives the energy metabolism of colonic epithelial cells toward β-oxidation and limits both the luminal bioavailability of oxygen and nitrate to prevent a dysbiotic expansion of potentially pathogenic *Escherichia* and *Salmonella*^92^. Mice lacking epithelial PPAR-γ signaling exhibited significantly elevated nitrate availability in the lumen and dysbiotic expansion of *Escherichia coli* that carries the genetic machinery for nitrate respiration^93^. Moreover, treatment with 5-amino salicylic acid, a PPAR-γ agonist, restored mitochondrial bioenergetics in the colonic epithelium, which alleviated dysbiosis triggered by high-fat diet and antibiotics^92^. A recent study has also established that intestinal hypoxia-inducible factor-2α (*Hif2α*) deficiency altered the balance of intestinal *Bacteroides vulgatus* to *Ruminococcus torques* to affect bile acid signaling and obesity-related insulin resistance^94^. Mechanistically, ablation of intestinal *Hif2α* reduced the level of epithelial-derived lactate, which enriched the polysaccharide utilization genes to promote the growth of *Bacteroides vulgatus*.

Host genetics can also shape the commensal microorganisms by regulating the secretion of defense signals, such as antimicrobial peptides and immunoglobulin A (IgA)^95^. Typically, secretory IgA directed against bacterial antigens has been proposed to shape intestinal microbial composition by multiple mechanisms, including inhibition of bacterial motility and reduction in bacterial fitness. As has been recently reported, activation of mechanistic target of rapamycin complex 1 (mTORC1) signal in CD11c cells alters IgA secretion at the mucosal site^96^. Reduced IgA production results in decreased gastrointestinal colonization of *Lactobacillus johnsonii* Q1–7, which in turn leads to lower food intake and body mass. In addition, another investigation has shown that mice with T cell-specific ablation of *Myd88*, an innate adaptor molecule, have defective development of T follicular helper cells in the gut, contributing to inappropriate IgA targeting of *Clostridia* and altered microbial balance^97^. This results in imbalanced expansion of *Desulfovibrio* at the expense of the loss of *Clostridia*, which contributes to increased lipid absorption and obesity susceptibility.

Goblet cell secretion of mucin represents another way by which host genetics influence the symbiotic microbes^98^. A recent study shows that loss of forkhead box O 1(*Foxo1*) in intestinal epithelial cells results in defects in goblet cell autophagy and mucus secretion, which induces gut dysbiosis, disruption of gut barrier integrity, and increased susceptibility to intestinal inflammation^99^. Deficiency G protein-coupled receptor 35 (*Gpr35*), previously known to regulate the activity of Na/K-ATPase and mitochondrial oxidative phosphorylation (OXPHOS) of epithelial cells^100^, was recently shown to induce goblet cell depletion and dysbiosis^101,102^. Interestingly, ablation of Calcitonin gene-related peptide (*Cgrp*) in nociceptor neurons or epithelial *Ramp1* is also shown to reduce goblet cell mucus secretion, which induces dysbiosis and susceptibility to colitis^103^.

## Practical considerations in gene-microbe-behavior study

The interwoven gene-microbiota connection in neurobehavioral changes, as well as the limitations in current techniques, suggests some practical issues deserving attention in the effort to better understand the etiology and treatment strategy for neuropsychiatric disease. Firstly, given the strong impact of environmental factors on microbial composition, diet composition, and housing condition variance should be carefully considered in screening differential bacteria^104^. Conclusive evidence that a genomic mutation is responsible for a specific behavioral phenotype can be obtained when littermate controls are used^21^. If wild-type control mice were provided from other animal facility, the influence of environment factors on gut flora was difficult to exclude. Of note, the fact that mice from different suppliers showed markedly variance in microbial background and biologic responses provides excellent clues for identifying key causal microbe, as demonstrated previously^61,105^.

Secondly, in mono-colonization studies, it would be necessary to profile whether the global microbial community would be affected to understand whether the host impact may derive from microbe interactions. Indeed, studies have shown that colonization of a single strain may elicit microbiome-wide changes^35^. It is reasonable that host impacts of gut microbial change could be attributed to the interactions among different microbes. Indeed, in the study of Miyauchi *et al*., mice mono-colonized with OTU0002 exhibited EAE symptoms that were less severe than those of specific-pathogen-free (SPF) mice, which indicated that other bacterial members of the microbiota could also be participating in the pathogenesis of EAE. In the search for potential microbial interactions, they further showed that two distinct signals from OTU0002 and a *L. reuteri* strain (H4 and LMG 18,238) coordinately activate autoreactive T cells in the small intestine to drive autoimmune responses. In practice, to better mimic microbial interaction network in host regulation, a simplified microbial community consisting of representative high-abundance bacterial species would provide more physiologically similar, while structurally defined microbial models for the validation of key causal microbes. This strategy is readily found in the study of Lobel *et al*.^106^ and Kasahara *et al*.^107^, which support that the colonization of the structurally defined consortium of bacteria could provide physiologically relevant and therefore more strong insights into microbe-host interactions.

Thirdly, although we largely focus on data from mice models, non-mouse model organisms, such as zebrafish, *Drosophila melanogaster, and Caenorhabditis elegans* are useful for gut-brain-microbiota studies related to neuropsychiatric diseases^108^. The accumulated expertise in genetic and microbial manipulation and human-relevant neurobehavioral parameters could become advantages for gaining holistic insights into gene-microbiota-behavior interplay and the signaling basis. Some elegant studies are available in relation to the dissection of microbial connection to sensory and locomotive behavior^83,109^.

Lastly, in the clinical setting, age, sex, dietary habits, co-morbid diseases, and drug exposure are common confounding factors for identifying causal links. Addressing these inter-individual variability factors is crucial to elucidating the genetic and microbial factors that causally shape human neuropsychiatry diseases. A recent prospective large-population study dissecting the influence of the gut microbiome, diet, and genetics on plasma metabolome variation presents a useful workflow to tackle this issue^110^. In particular, through Mendelian randomization and mediation analyses, this study uncovered putative causal relationships between the gut microbiome and plasma metabolites. Application to neuropsychiatric disorders would be expected to clarify robust microbial features to clinical diagnostic and preventive purposes.

## Conclusions

Current therapeutic approaches for neuropsychiatric disorders aim to target the brain directly. The accumulating findings on host genetic-gut microbial interplay in neuropsychiatric diseases broaden our views on the factors shaping individual’s susceptibility to mental illnesses and underscore the bidirectional gut-brain crosstalk in behavioral control. An emerging picture is that microbial and host genetic factors may control different aspects of brain diseases, which provide the rationale for combinatorial targeting of genetic factors and microbial signaling for comprehensive intervention of complex behavioral diseases. Deciphering gene-microbiome-phenotype trialogue in complex neuropsychiatric diseases may therefore inspire novel therapeutic strategies and precision intervention. However, since most of the causal data are from animal studies, the results still should be approximated to humans with caution.

The field of microbiome research is experiencing a progression from associative linking to causal and mechanistic insights, which presents new opportunities to decipher how this dynamic community is functionally implicated in the genetic predisposition to neuropsychiatric diseases in concert with other environmental factors such as diet and lifestyle. In future studies, the causal relationship between genetic variance, key microorganisms, and behavioral changes remains to be fully elucidated in the clinical setting in order to provide novel diagnostic biomarkers and therapeutic targets. Another key question is how the microbes achieve central relays of local signals to specify the behavioral changes. Research insights into the communication pathways between the gut and the brain is critical to drive this frontier forward. Meanwhile, elucidation of the key biologic synthetic pathways of microbes and the sensors for the signaling molecules could provide potential drug targets. Recently, the devise of novel techniques for genetic manipulation of gut microbes and targeted protein degradation in bacteria could enable efficient clues to answer these key questions^111,112^. Neural circuitry allowing gut microbial control over behaviors remains to be fully unmapped, under both physiological and pathological conditions. Advances in this field increasingly benefit from the technical advances in optogenetics, chemogenetics, and virally delivered molecular tools for neural manipulation in an organ-specific and inter-organ manner^71,113^. Progress in this field could be integrated with genetic manipulation studies to offer combinatory targets for future drug discovery. Specifically, the microbial signals or microbe-responsive pathways promoting inner homeostasis or resilience to stressful events could be strengthened as a preventive or therapeutic strategy.

## Data Availability

Data sharing is not applicable to this article as no datasets were generated or analyzed during the current study.

## References

[1] An JY, Lin K, Zhu L, Werling DM, Dong S, Brand H, Wang HZ, Zhao X, Schwartz GB, Collins RL, et al. Genome-wide de novo risk score implicates promoter variation in autism spectrum disorder. Science. 2018;362(6420):6420. doi:10.1126/science.aat6576. PMC6432922 30545852

[2] Levey DF, Stein MB, Wendt FR, Pathak GA, Zhou H, Aslan M, Quaden R, Harrington KM, Nuñez YZ, Overstreet C, et al. Bi-ancestral depression GWAS in the million veteran program and meta-analysis in >1.2 million individuals highlight new therapeutic directions. Nat Neurosci. 2021;24(7):954–18. doi:10.1038/s41593-021-00860-2. 34045744 PMC8404304

[3] Bipolar D, Ripke S, McQuillin A, Boocock J, Stahl EA, Pavlides JMW, Mullins N, Charney AW, Ori APS, Loohuis LMO, et al. Electronic address, D bipolar, and C schizophrenia working group of the psychiatric genomics, genomic dissection of bipolar disorder and schizophrenia, including 28 subphenotypes. Cell. 2018;173(7):1705–1715 e16. doi:10.1016/j.cell.2018.05.046. 29906448 PMC6432650

[4] Grove J, Ripke S, Als TD, Mattheisen M, Walters RK, Won H, Pallesen J, Agerbo E, Andreassen OA, Anney R, et al. Identification of common genetic risk variants for autism spectrum disorder. Nat Genet. 2019;51(3):431–444. doi:10.1038/s41588-019-0344-8. 30804558 PMC6454898

[5] Ni JJ, Xu Q, Yan SS, Han BX, Zhang H, Wei XT, Feng GJ, Zhao M, Pei YF, Zhang L. Gut microbiota and psychiatric disorders: a two-sample Mendelian randomization study. Front Microbiol. 2021;12:737197. doi:10.3389/fmicb.2021.737197. 35185808 PMC8856606

[6] Ma B, Liang J, Dai M, Wang J, Luo J, Zhang Z, Jing J. Altered gut microbiota in Chinese children with autism spectrum disorders. Front Cell Infect Microbiol. 2019;9:40. doi:10.3389/fcimb.2019.00040. 30895172 PMC6414714

[7] Strati F, Cavalieri D, Albanese D, De Felice C, Donati C, Hayek J, Jousson O, Leoncini S, Renzi D, Calabro A, et al. New evidences on the altered gut microbiota in autism spectrum disorders. Microbiome. 2017;5(1):24. doi:10.1186/s40168-017-0242-1. 28222761 PMC5320696

[8] Valles-Colomer M, Falony G, Darzi Y, Tigchelaar EF, Wang J, Tito RY, Schiweck C, Kurilshikov A, Joossens M, Wijmenga C, et al. The neuroactive potential of the human gut microbiota in quality of life and depression. Nat Microbiol. 2019;4(4):623–632. doi:10.1038/s41564-018-0337-x. 30718848

[9] Levey DF, Stein MB, Wendt FR, Pathak GA, Zhou H, Aslan M, Quaden R, Harrington KM, Nunez YZ, Overstreet C, et al. Bi-ancestral depression GWAS in the million veteran program and meta-analysis in >1.2 million individuals highlight new therapeutic directions. Nat Neurosci. 2021;24(7):954–963. doi:10.1038/s41593-021-00860-2. 34045744 PMC8404304

[10] Zhuang Z, Yang R, Wang W, Qi L, Huang T. Associations between gut microbiota and Alzheimer’s disease, major depressive disorder, and schizophrenia. J Neuroinflammation. 2020;17(1):288. doi:10.1186/s12974-020-01961-8. 33008395 PMC7532639

[11] Li HJ, Zhang C, Hui L, Zhou DS, Li Y, Zhang CY, Wang C, Wang L, Li W, Yang Y, et al. Novel risk loci associated with genetic risk for bipolar disorder among han Chinese individuals: a genome-wide association study and meta-analysis. JAMA Psychiatry. 2021;78(3):320–330. doi:10.1001/jamapsychiatry.2020.3738. 33263727 PMC7711567

[12] Mullins N, Forstner AJ, O’Connell KS, Coombes B, Coleman JRI, Qiao Z, Als TD, Bigdeli TB, Børte S, Bryois J, et al. Genome-wide association study of more than 40,000 bipolar disorder cases provides new insights into the underlying biology. Nat Genet. 2021;53(6):817–829. doi:10.1038/s41588-021-00857-4. 34002096 PMC8192451

[13] McGuinness AJ, Davis JA, Dawson SL, Loughman A, Collier F, O’Hely M, Simpson CA, Green J, Marx W, Hair C, et al. A systematic review of gut microbiota composition in observational studies of major depressive disorder, bipolar disorder and schizophrenia. Mol Psychiatry. 2022;27(4):1920–1935. doi:10.1038/s41380-022-01456-3. 35194166 PMC9126816

[14] Trubetskoy V, Pardiñas AF, Qi T, Panagiotaropoulou G, Awasthi S, Bigdeli TB, Bryois J, Chen CY, Dennison CA, Hall LS, et al. Mapping genomic loci implicates genes and synaptic biology in schizophrenia. Nature. 2022;604(7906):502–508. doi:10.1038/s41586-022-04434-5. 35396580 PMC9392466

[15] Lintas C, Sacco R, Persico AM. Genome-wide expression studies in autism spectrum disorder, Rett syndrome, and Down syndrome. Neurobiol Dis. 2012;45(1):57–68. doi:10.1016/j.nbd.2010.11.010. 21130877

[16] Borghi E, Borgo F, Severgnini M, Savini MN, Casiraghi MC, Vignoli A. Rett syndrome: a focus on gut microbiota. Int J Mol Sci. 2017;18(2):344. doi:10.3390/ijms18020344. 28178201 PMC5343879

[17] Harper CB, Smillie KJ. Current molecular approaches to investigate pre-synaptic dysfunction. J Neurochem. 2021;157(2):107–129. doi:10.1111/jnc.15316. 33544872

[18] Sullivan PF, Daly MJ, O’Donovan M. Genetic architectures of psychiatric disorders: the emerging picture and its implications. Nat Rev Genet. 2012;13(8):537–551. doi:10.1038/nrg3240. 22777127 PMC4110909

[19] Zhang Y, Fan Q, Hou Y, Zhang X, Yin Z, Cai X, Wei W, Wang J, He D, Wang G, et al. Bacteroides species differentially modulate depression-like behavior via gut-brain metabolic signaling. Brain Behav Immun. 2022;102:11–22. doi:10.1016/j.bbi.2022.02.007. 35143877

[20] Morais LH, Schreiber H, Mazmanian SK. The gut microbiota-brain axis in behaviour and brain disorders. Nat Rev Microbiol. 2021;19(4):241–255. doi:10.1038/s41579-020-00460-0. 33093662

[21] Buffington SA, Dooling SW, Sgritta M, Noecker C, Murillo OD, Felice DF, Turnbaugh PJ, Costa-Mattioli M. Dissecting the contribution of host genetics and the microbiome in complex behaviors. Cell. 2021;184(7):1740–1756.e16. doi:10.1016/j.cell.2021.02.009. 33705688 PMC8996745

[22] Vuong HE, Hsiao EY. Emerging roles for the gut microbiome in autism spectrum disorder. Biol Psychiatry. 2017;81(5):411–423. doi:10.1016/j.biopsych.2016.08.024. 27773355 PMC5285286

[23] Coury DL, Ashwood P, Fasano A, Fuchs G, Geraghty M, Kaul A, Mawe G, Patterson P, Jones NE. Gastrointestinal conditions in children with autism spectrum disorder: developing a research agenda. Pediatrics. 2012;130(Suppl 2):S160–8. doi:10.1542/peds.2012-0900N. 23118247

[24] Flowers SA, Ward KM, Clark CT. The gut microbiome in bipolar disorder and pharmacotherapy management. Neuropsychobiology. 2020;79(1):43–49. doi:10.1159/000504496. 31722343

[25] Black CJ, Drossman DA, Talley NJ, Ruddy J, Ford AC. Functional gastrointestinal disorders: advances in understanding and management. Lancet. 2020;396(10263):1664–1674. doi:10.1016/S0140-6736(20)32115-2. 33049221

[26] Yap CX, Henders AK, Alvares GA, Wood DLA, Krause L, Tyson GW, Restuadi R, Wallace L, McLaren T, Hansell NK, et al. Autism-related dietary preferences mediate autism-gut microbiome associations. Cell. 2021;184(24):5916–5931 e17. doi:10.1016/j.cell.2021.10.015. 34767757

[27] Yu Y, Zhao F. Microbiota-gut-brain axis in autism spectrum disorder. J Genet Genomics. 2021;48(9):755–762. doi:10.1016/j.jgg.2021.07.001. 34373221

[28] Lou M, Cao A, Jin C, Mi K, Xiong X, Zeng Z, Pan X, Qie J, Qiu S, Niu Y, et al. Deviated and early unsustainable stunted development of gut microbiota in children with autism spectrum disorder. Gut. 2021. doi:10.1136/gutjnl-2021-325115. PMC9279844 34930815

[29] Pfaender S, Sauer AK, Hagmeyer S, Mangus K, Linta L, Liebau S, Bockmann J, Huguet G, Bourgeron T, Boeckers TM, et al. Zinc deficiency and low enterocyte zinc transporter expression in human patients with autism related mutations in SHANK3. Sci Rep. 2017;7(1):45190. doi:10.1038/srep45190. 28345660 PMC5366950

[30] Katayama Y, Nishiyama M, Shoji H, Ohkawa Y, Kawamura A, Sato T, Suyama M, Takumi T, Miyakawa T, Nakayama KI. CHD8 haploinsufficiency results in autistic-like phenotypes in mice. Nature. 2016;537(7622):675–679. doi:10.1038/nature19357. 27602517

[31] Liyanage VR, Rastegar M. Rett syndrome and MeCP2. Neuromolecular Med. 2014;16(2):231–264. doi:10.1007/s12017-014-8295-9. 24615633 PMC5798978

[32] Strati F, Cavalieri D, Albanese D, De Felice C, Donati C, Hayek J, Jousson O, Leoncini S, Pindo M, Renzi D, et al. Altered gut microbiota in Rett syndrome. Microbiome. 2016;4(1):41. doi:10.1186/s40168-016-0185-y. 27473171 PMC4967335

[33] Palepu MSK, Dandekar MP. Remodeling of microbiota gut-brain axis using psychobiotics in depression. Eur J Pharmacol. 2022;931:175171. doi:10.1016/j.ejphar.2022.175171. 35926568

[34] Yu Y, Zhang B, Ji P, Zuo Z, Huang Y, Wang N, Liu C, Liu SJ, Zhao F. Changes to gut amino acid transporters and microbiome associated with increased E/I ratio in Chd8(±) mouse model of ASD-like behavior. Nat Commun. 2022;13(1):1151. doi:10.1038/s41467-022-28746-2. 35241668 PMC8894489

[35] Sgritta M, Dooling SW, Buffington SA, Momin EN, Francis MB, Britton RA, Costa-Mattioli M. Mechanisms underlying microbial-mediated changes in social behavior in mouse models of autism spectrum disorder. Neuron. 2019;101(2):246–259.e6. doi:10.1016/j.neuron.2018.11.018. 30522820 PMC6645363

[36] Tabouy L, Getselter D, Ziv O, Karpuj M, Tabouy T, Lukic I, Maayouf R, Werbner N, Ben-Amram H, Nuriel-Ohayon M, et al. Dysbiosis of microbiome and probiotic treatment in a genetic model of autism spectrum disorders. Brain Behav Immun. 2018;73:310–319. doi:10.1016/j.bbi.2018.05.015. 29787855

[37] Li Y, Luo ZY, Hu YY, Bi YW, Yang JM, Zou WJ, Song YL, Li S, Shen T, Li SJ, et al. The gut microbiota regulates autism-like behavior by mediating vitamin B(6) homeostasis in EphB6-deficient mice. Microbiome. 2020;8(1):120. doi:10.1186/s40168-020-00884-z. 32819434 PMC7441571

[38] Zhang Y, Huang R, Cheng M, Wang L, Chao J, Li J, Zheng P, Xie P, Zhang Z, Yao H. Gut microbiota from NLRP3-deficient mice ameliorates depressive-like behaviors by regulating astrocyte dysfunction via circHIPK2. Microbiome. 2019;7(1):116. doi:10.1186/s40168-019-0733-3. 31439031 PMC6706943

[39] Wang S, Ishima T, Qu Y, Shan J, Chang L, Wei Y, Zhang J, Pu Y, Fujita Y, Tan Y, et al. Ingestion of faecalibaculum rodentium causes depression-like phenotypes in resilient Ephx2 knock-out mice: a role of brain–gut–microbiota axis via the subdiaphragmatic vagus nerve. J Affect Disord. 2021;292:565–573. doi:10.1016/j.jad.2021.06.006. 34147969 PMC8282729

[40] Pu Y, Tan Y, Qu Y, Chang L, Wang S, Wei Y, Wang X, Hashimoto K. A role of the subdiaphragmatic vagus nerve in depression-like phenotypes in mice after fecal microbiota transplantation from Chrna7 knock-out mice with depression-like phenotypes. Brain Behav Immun. 2021;94:318–326. doi:10.1016/j.bbi.2020.12.032. 33422641

[41] Sauer AK, Bockmann J, Steinestel K, Boeckers TM, Grabrucker AM. Altered intestinal morphology and microbiota composition in the autism spectrum disorders associated SHANK3 mouse model. Int J Mol Sci. 2019;20(9):2134. doi:10.3390/ijms20092134. 31052177 PMC6540607

[42] Gallucci A, Patterson KC, Weit AR, Van Der Pol WJ, Dubois LG, Percy AK, Morrow CD, Campbell SL, Olsen ML. Microbial community changes in a female rat model of Rett syndrome. Prog Neuropsychopharmacol Biol Psychiatry. 2021;109:110259. doi:10.1016/j.pnpbp.2021.110259. 33548354 PMC8724884

[43] Sudo N, Chida Y, Aiba Y, Sonoda J, Oyama N, Yu XN, Kubo C, Koga Y. Postnatal microbial colonization programs the hypothalamic-pituitary-adrenal system for stress response in mice. J Physiol. 2004;558(1):263–275. doi:10.1113/jphysiol.2004.063388. 15133062 PMC1664925

[44] O’Mahony SM, Marchesi JR, Scully P, Codling C, Ceolho AM, Quigley EM, Cryan JF, Dinan TG. Early life stress alters behavior, immunity, and microbiota in rats: implications for irritable bowel syndrome and psychiatric illnesses. Biol Psychiatry. 2009;65(3):263–267. doi:10.1016/j.biopsych.2008.06.026. 18723164

[45] Li W, Dowd SE, Scurlock B, Acosta-Martinez V, Lyte M. Memory and learning behavior in mice is temporally associated with diet-induced alterations in gut bacteria. Physiol Behav. 2009;96(4–5):557–567. doi:10.1016/j.physbeh.2008.12.004. 19135464

[46] MacFabe DF, Cain NE, Boon F, Ossenkopp KP, Cain DP. Effects of the enteric bacterial metabolic product propionic acid on object-directed behavior, social behavior, cognition, and neuroinflammation in adolescent rats: relevance to autism spectrum disorder. Behav Brain Res. 2011;217(1):47–54. doi:10.1016/j.bbr.2010.10.005. 20937326

[47] Hsiao EY, McBride SW, Hsien S, Sharon G, Hyde ER, McCue T, Codelli JA, Chow J, Reisman SE, Petrosino JF, et al. Microbiota modulate behavioral and physiological abnormalities associated with neurodevelopmental disorders. Cell. 2013;155(7):1451–1463. doi:10.1016/j.cell.2013.11.024. 24315484 PMC3897394

[48] Desbonnet L, Clarke G, Shanahan F, Dinan TG, Cryan JF. Microbiota is essential for social development in the mouse. Mol Psychiatry. 2014;19(2):146–148. doi:10.1038/mp.2013.65. 23689536 PMC3903109

[49] Dowling LR, Strazzari MR, Keely S, Kaiko GE. Enteric nervous system and intestinal epithelial regulation of the gut-brain axis. J Allergy Clin Immunol. 2022;150(3):513–522. doi:10.1016/j.jaci.2022.07.015. 36075637

[50] Sharon G, Cruz NJ, Kang DW, Gandal MJ, Wang B, Kim YM, Zink EM, Casey CP, Taylor BC, Lane CJ, et al. Human gut microbiota from autism spectrum disorder promote behavioral symptoms in mice. Cell. 2019;177(6):1600–1618.e17. doi:10.1016/j.cell.2019.05.004. 31150625 PMC6993574

[51] Lichtenstein P, Yip BH, Bjork C, Pawitan Y, Cannon TD, Sullivan PF, Hultman CM. Common genetic determinants of schizophrenia and bipolar disorder in Swedish families: a population-based study. Lancet. 2009;373(9659):234–239. doi:10.1016/S0140-6736(09)60072-6. 19150704 PMC3879718

[52] Sublette ME, Cheung S, Lieberman E, Hu S, Mann JJ, Uhlemann AC, Miller JM. Bipolar disorder and the gut microbiome: a systematic review. Bipolar Disord. 2021;23(6):544–564. doi:10.1111/bdi.13049. 33512753

[53] Aizawa E, Tsuji H, Asahara T, Takahashi T, Teraishi T, Yoshida S, Koga N, Hattori K, Ota M, Kunugi H. Bifidobacterium and lactobacillus counts in the gut microbiota of patients with bipolar disorder and healthy controls. Front Psychiatry. 2018;9:730. doi:10.3389/fpsyt.2018.00730. 30713509 PMC6346636

[54] Zhu F, Ju Y, Wang W, Wang Q, Guo R, Ma Q, Sun Q, Fan Y, Xie Y, Yang Z, et al. Metagenome-wide association of gut microbiome features for schizophrenia. Nat Commun. 2020;11(1):1612. doi:10.1038/s41467-020-15457-9. 32235826 PMC7109134

[55] Altimiras F, Garcia JA, Palacios-García I, Hurley MJ, Deacon R, González B, Cogram P. Altered gut microbiota in a fragile X syndrome mouse model. Front Neurosci. 2021;15:653120. doi:10.3389/fnins.2021.653120. 34121987 PMC8190892

[56] Goo N, Bae HJ, Park K, Kim J, Jeong Y, Cai M, Cho K, Jung SY, Kim DH, Ryu JH. The effect of fecal microbiota transplantation on autistic-like behaviors in Fmr1 KO mice. Life Sci. 2020;262:118497. doi:10.1016/j.lfs.2020.118497. 32987062

[57] An R, Wu Y, Li Y, Li X, Ai S, Xu Y, He C. Pain-related factors and their impact on quality of life in Chinese patients with amyotrophic lateral sclerosis. Front Neurosci. 2022;16:897598. doi:10.3389/fnins.2022.897598. 35924224 PMC9340542

[58] Mejzini R, Flynn LL, Pitout IL, Fletcher S, Wilton SD, Akkari PA. ALS genetics, mechanisms, and therapeutics: where are we now? Front Neurosci. 2019;13:1310. doi:10.3389/fnins.2019.01310. 31866818 PMC6909825

[59] Zhang YG, Wu S, Yi J, Xia Y, Jin D, Zhou J, Sun J. Target intestinal microbiota to alleviate disease progression in amyotrophic lateral sclerosis. Clin Ther. 2017;39(2):322–336. doi:10.1016/j.clinthera.2016.12.014. 28129947 PMC5344195

[60] Blacher E, Bashiardes S, Shapiro H, Rothschild D, Mor U, Dori-Bachash M, Kleimeyer C, Moresi C, Harnik Y, Zur M, et al. Potential roles of gut microbiome and metabolites in modulating ALS in mice. Nature. 2019;572(7770):474–480. doi:10.1038/s41586-019-1443-5. 31330533

[61] Burberry A, Wells MF, Limone F, Couto A, Smith KS, Keaney J, Gillet G, van Gastel N, Wang JY, Pietilainen O, et al. C9orf72 suppresses systemic and neural inflammation induced by gut bacteria. Nature. 2020;582(7810):89–94. doi:10.1038/s41586-020-2288-7. 32483373 PMC7416879

[62] Faith JJ, Ahern PP, Ridaura VK, Cheng J, Gordon JI. Identifying gut microbe-host phenotype relationships using combinatorial communities in gnotobiotic mice. Sci Transl Med. 2014;6(220):220ra11. doi:10.1126/scitranslmed.3008051. PMC3973144 24452263

[63] Surana NK, Kasper DL. Moving beyond microbiome-wide associations to causal microbe identification. Nature. 2017;552(7684):244–247. doi:10.1038/nature25019. 29211710 PMC5730484

[64] Regen T, Isaac S, Amorim A, Núñez NG, Hauptmann J, Shanmugavadivu A, Klein M, Sankowski R, Mufazalov IA, Yogev N, et al. 2021. IL-17 controls central nervous system autoimmunity through the intestinal microbiome. Sci Immunol. 6(56). doi:10.1126/sciimmunol.aaz6563. 33547052

[65] Elinav E, Strowig T, Kau AL, Henao-Mejia J, Thaiss CA, Booth CJ, Peaper DR, Bertin J, Eisenbarth SC, Gordon JI, et al. NLRP6 inflammasome regulates colonic microbial ecology and risk for colitis. Cell. 2011;145(5):745–757. doi:10.1016/j.cell.2011.04.022. 21565393 PMC3140910

[66] Rosen CE, Palm NW. Navigating the microbiota seas: triangulation finds a way forward. Cell Host & Microbe. 2018;23(1):1–3. doi:10.1016/j.chom.2017.12.015. 29324223

[67] Guo H, Chou WC, Lai Y, Liang K, Tam JW, Brickey WJ, Chen L, Montgomery ND, Li X, Bohannon LM, et al. Multi-omics analyses of radiation survivors identify radioprotective microbes and metabolites. Science. 2020;370(6516):6516. doi:10.1126/science.aay9097. PMC7898465 33122357

[68] Miyauchi E, Kim SW, Suda W, Kawasumi M, Onawa S, Taguchi-Atarashi N, Morita H, Taylor TD, Hattori M, Ohno H. Gut microorganisms act together to exacerbate inflammation in spinal cords. Nature. 2020;585(7823):102–106. doi:10.1038/s41586-020-2634-9. 32848245

[69] Wu WL, Adame MD, Liou CW, Barlow JT, Lai TT, Sharon G, Schretter CE, Needham BD, Wang MI, Tang W, et al. Microbiota regulate social behaviour via stress response neurons in the brain. Nature. 2021;595(7867):409–414. doi:10.1038/s41586-021-03669-y. 34194038 PMC8346519

[70] Mayneris-Perxachs J, Castells-Nobau A, Arnoriaga-Rodriguez M, Martin M, de la Vega-Correa L, Zapata C, Burokas A, Blasco G, Coll C, Escrichs A, et al. Microbiota alterations in proline metabolism impact depression. Cell Metab. 2022;34(5):681–701 e10. doi:10.1016/j.cmet.2022.04.001. 35508109

[71] Mars RAT, Yang Y, Ward T, Houtti M, Priya S, Lekatz HR, Tang X, Sun Z, Kalari KR, Korem T, et al. Longitudinal multi-omics reveals subset-specific mechanisms underlying irritable bowel syndrome. Cell. 2020;182(6):1460–1473.e17. doi:10.1016/j.cell.2020.08.007. 32916129 PMC8109273

[72] Schluter J, Peled JU, Taylor BP, Markey KA, Smith M, Taur Y, Niehus R, Staffas A, Dai A, Fontana E, et al. The gut microbiota is associated with immune cell dynamics in humans. Nature. 2020;588(7837):303–307. doi:10.1038/s41586-020-2971-8. 33239790 PMC7725892

[73] Xiao LW, Zhang FY, Zhao FQ. Large-scale microbiome data integration enables robust biomarker identification. Nat Comput Sci. 2022;2(5):307–316. doi:10.1038/s43588-022-00247-8. PMC10766547 38177817

[74] Priya S, Burns MB, Ward T, Mars RAT, Adamowicz B, Lock EF, Kashyap PC, Knights D, Blekhman R. Identification of shared and disease-specific host gene-microbiome associations across human diseases using multi-omic integration. Nat Microbiol. 2022;7(6):780–795. doi:10.1038/s41564-022-01121-z. 35577971 PMC9159953

[75] Leonard I, Gao IH, Lin WY, Allen M, Li XV, Fiers WD, Celie MD, Putzel GG, Yantiss RK, Johncilla M, et al. Mucosal fungi promote gut barrier function and social behavior via type 17 immunity. Cell. 2022;185(5):831–846.e14. doi:10.1016/j.cell.2022.01.017. 35176228 PMC8897247

[76] Gershon MD, Margolis KG. The gut, its microbiome, and the brain: connections and communications. J Clin Invest. 2021 ;131(18). doi:10.1172/JCI143768. PMC8439601 34523615

[77] Bravo JA, Forsythe P, Chew MV, Escaravage E, Savignac HM, Dinan TG, Bienenstock J, Cryan JF. Ingestion of lactobacillus strain regulates emotional behavior and central GABA receptor expression in a mouse via the vagus nerve. Proc Natl Acad Sci U S A. 2011;108(38):16050–16055. doi:10.1073/pnas.1102999108. 21876150 PMC3179073

[78] Muller PA, Schneeberger M, Matheis F, Wang P, Kerner Z, Ilanges A, Pellegrino K, Del Mármol J, Castro TBR, Furuichi M, et al. Microbiota modulate sympathetic neurons via a gut–brain circuit. Nature. 2020;583(7816):441–446. doi:10.1038/s41586-020-2474-7. 32641826 PMC7367767

[79] Kaelberer MM, Buchanan KL, Klein ME, Barth BB, Montoya MM, Shen X, Bohórquez DV. A gut-brain neural circuit for nutrient sensory transduction. Science. 2018;361(6408):6408. doi:10.1126/science.aat5236. PMC6417812 30237325

[80] Ye L, Bae M, Cassilly CD, Jabba SV, Thorpe DW, Martin AM, Lu HY, Wang J, Thompson JD, Lickwar CR, et al. Enteroendocrine cells sense bacterial tryptophan catabolites to activate enteric and vagal neuronal pathways. Cell Host & Microbe. 2021;29(2):179–196 e9. doi:10.1016/j.chom.2020.11.011. 33352109 PMC7997396

[81] Needham BD, Funabashi M, Adame MD, Wang Z, Boktor JC, Haney J, Wu WL, Rabut C, Ladinsky MS, Hwang SJ, et al. A gut-derived metabolite alters brain activity and anxiety behaviour in mice. Nature. 2022;602(7898):647–653. doi:10.1038/s41586-022-04396-8. 35165440 PMC9170029

[82] Gabanyi I, Lepousez G, Wheeler R, Vieites-Prado A, Nissant A, Wagner S, Moigneu C, Dulauroy S, Hicham S, Polomack B, et al. Bacterial sensing via neuronal Nod2 regulates appetite and body temperature. Science. 2022;376(6590):eabj3986. doi:10.1126/science.abj3986. 35420957

[83] Schretter CE, Vielmetter J, Bartos I, Marka Z, Marka S, Argade S, Mazmanian SK. A gut microbial factor modulates locomotor behaviour in Drosophila. Nature. 2018;563(7731):402–406. doi:10.1038/s41586-018-0634-9. 30356215 PMC6237646

[84] Zhang T, Perkins MH, Chang H, Han W, de Araujo IE. An inter-organ neural circuit for appetite suppression. Cell. 2022; 185 (14): 2478–2494. doi:10.1016/j.cell.2022.05.007 . 35662413 PMC9433108

[85] Muscogiuri G, DeFronzo RA, Gastaldelli A, Holst JJ. Glucagon-like peptide-1 and the central/peripheral nervous system: crosstalk in diabetes. Trends Endocrinol Metab. 2017;28(2):88–103. doi:10.1016/j.tem.2016.10.001. 27871675

[86] Medina-Rodriguez EM, Madorma D, O’Connor G, Mason BL, Han D, Deo SK, Oppenheimer M, Nemeroff CB, Trivedi MH, Daunert S, et al. Identification of a signaling mechanism by which the microbiome regulates Th17 cell-mediated depressive-like behaviors in mice. Am J Psychiatry. 2020;177(10):974–990. doi:10.1176/appi.ajp.2020.19090960. 32731813 PMC7647050

[87] Alves de Lima K, Rustenhoven J, Da Mesquita S, Wall M, Salvador AF, Smirnov I, Martelossi Cebinelli G, Mamuladze T, Baker W, Papadopoulos Z, et al. Meningeal γδ T cells regulate anxiety-like behavior via IL-17a signaling in neurons. Nat Immunol. 2020;21(11):1421–1429. doi:10.1038/s41590-020-0776-4. 32929273 PMC8496952

[88] Kim S, Kim H, Yim YS, Ha S, Atarashi K, Tan TG, Longman RS, Honda K, Littman DR, Choi GB, et al. Maternal gut bacteria promote neurodevelopmental abnormalities in mouse offspring. Nature. 2017;549(7673):528–532. doi:10.1038/nature23910. 28902840 PMC5870873

[89] Reed MD, Yim YS, Wimmer RD, Kim H, Ryu C, Welch GM, Andina M, King HO, Waisman A, Halassa MM, et al. IL-17a promotes sociability in mouse models of neurodevelopmental disorders. Nature. 2020;577(7789):249–253. doi:10.1038/s41586-019-1843-6. 31853066 PMC8112727

[90] Benson AK, Kelly SA, Legge R, Ma F, Low SJ, Kim J, Zhang M, Oh PL, Nehrenberg D, Hua K, et al. Individuality in gut microbiota composition is a complex polygenic trait shaped by multiple environmental and host genetic factors. Proc Natl Acad Sci U S A. 2010;107(44):18933–18938. doi:10.1073/pnas.1007028107. 20937875 PMC2973891

[91] Goodrich JK, Waters JL, Poole AC, Sutter JL, Koren O, Blekhman R, Beaumont M, Van Treuren W, Knight R, Bell JT, et al. Human genetics shape the gut microbiome. Cell. 2014;159(4):789–799. doi:10.1016/j.cell.2014.09.053. 25417156 PMC4255478

[92] Lee JY, Cevallos SA, Byndloss MX, Tiffany CR, Olsan EE, Butler BP, Young BM, Rogers AWL, Nguyen H, Kim K, et al. High-fat diet and antibiotics cooperatively impair mitochondrial bioenergetics to trigger dysbiosis that exacerbates pre-inflammatory bowel disease. Cell Host & Microbe. 2020;28(2):273–284.e6. doi:10.1016/j.chom.2020.06.001. 32668218 PMC7429289

[93] Byndloss MX, Olsan EE, Rivera-Chávez F, Tiffany CR, Cevallos SA, Lokken KL, Torres TP, Byndloss AJ, Faber F, Gao Y, et al. Microbiota-activated PPAR-γ signaling inhibits dysbiotic Enterobacteriaceae expansion. Science. 2017;357(6351):570–575. doi:10.1126/science.aam9949. 28798125 PMC5642957

[94] Wu Q, Liang X, Wang K, Lin J, Wang X, Wang P, Zhang Y, Nie Q, Liu H, Zhang Z, et al. Intestinal hypoxia-inducible factor 2α regulates lactate levels to shape the gut microbiome and alter thermogenesis. Cell Metab. 2021;33(10):1988–2003 e7. doi:10.1016/j.cmet.2021.07.007. 34329568

[95] Kubinak JL, Petersen C, Stephens WZ, Soto R, Bake E, O’Connell RM, Round JL. MyD88 signaling in T cells directs IgA-Mediated control of the microbiota to promote health. Cell Host & Microbe. 2015;17(2):153–163. doi:10.1016/j.chom.2014.12.009. 25620548 PMC4451207

[96] Chagwedera DN, Ang QY, Bisanz JE, Leong YA, Ganeshan K, Cai J, Patterson AD, Turnbaugh PJ, Chawla A. Nutrient sensing in CD11c cells alters the gut microbiota to regulate food intake and body mass. Cell Metab. 2019;30(2):364–373.e7. doi:10.1016/j.cmet.2019.05.002. 31130466 PMC6687538

[97] Petersen C, Bell R, Klag KA, Lee SH, Soto R, Ghazaryan A, Buhrke K, Ekiz HA, Ost KS, Boudina S, et al. T cell–mediated regulation of the microbiota protects against obesity. Science. 2019;365(6451):6451. doi:10.1126/science.aat9351. PMC7294966 31346040

[98] Zhang J, Yu Q, Jiang D, Yu K, Yu W, Chi Z, Chen S, Li M, Yang D, Wang Z, et al. Epithelial gasdermin D shapes the host-microbial interface by driving mucus layer formation. Sci Immunol. 2022;7(68):eabk2092. doi:10.1126/sciimmunol.abk2092. 35119941

[99] Wu N, Sun H, Zhao X, Zhang Y, Tan J, Qi Y, Wang Q, Ng M, Liu Z, He L, et al. MAP3K2-regulated intestinal stromal cells define a distinct stem cell niche. Nature. 2021;592(7855):606–610. doi:10.1038/s41586-021-03283-y. 33658717

[100] Schneditz G, Elias JE, Pagano E, Zaeem Cader M, Saveljeva S, Long K, Mukhopadhyay S, Arasteh M, Lawley TD, Dougan G, et al. 2019. GPR35 promotes glycolysis, proliferation, and oncogenic signaling by engaging with the sodium potassium pump. Sci Signal. 12(562). doi:10.1126/scisignal.aau9048. PMC6364804 30600262

[101] Melhem H, Kaya B, Kaymak T, Wuggenig P, Flint E, Roux J, Oost KC, Cavelti-Weder C, Balmer ML, Walser JC, et al. Epithelial GPR35 protects from Citrobacter rodentium infection by preserving goblet cells and mucosal barrier integrity. Mucosal Immunol. 2022;15(3):443–458. doi:10.1038/s41385-022-00494-y. 35264769 PMC9038528

[102] Wu X, Chen S, Yan Q, Yu F, Shao H, Zheng X, Zhang X. Gpr35 shapes gut microbial ecology to modulate hepatic steatosis. Pharmacol Res. 2023;189:106690. doi:10.1016/j.phrs.2023.106690. 36758734

[103] Yang D, Jacobson A, Meerschaert KA, Sifakis JJ, Wu M, Chen X, Yang T, Zhou Y, Anekal PV, Rucker RA, et al. Nociceptor neurons direct goblet cells via a CGRP-RAMP1 axis to drive mucus production and gut barrier protection. Cell. 2022;185(22):4190–4205.e25. doi:10.1016/j.cell.2022.09.024. 36243004 PMC9617795

[104] Hou Y, Wei W, Guan X, Liu Y, Bian G, He D, Fan Q, Cai X, Zhang Y, Wang G, et al. A diet-microbial metabolism feedforward loop modulates intestinal stem cell renewal in the stressed gut. Nat Commun. 2021;12(1):271. doi:10.1038/s41467-020-20673-4. 33431867 PMC7801547

[105] Sivan A, Corrales L, Hubert N, Williams JB, Aquino-Michaels K, Earley ZM, Benyamin FW, Lei YM, Jabri B, Alegre ML, et al. Commensal Bifidobacterium promotes antitumor immunity and facilitates anti–PD-L1 efficacy. Science. 2015;350(6264):1084–1089. doi:10.1126/science.aac4255. 26541606 PMC4873287

[106] Lobel L, Cao YG, Fenn K, Glickman JN, Garrett WS. Diet posttranslationally modifies the mouse gut microbial proteome to modulate renal function. Science. 2020;369(6510):1518–1524. doi:10.1126/science.abb3763. 32943527 PMC8178816

[107] Kasahara K, Krautkramer KA, Org E, Romano KA, Kerby RL, Vivas EI, Mehrabian M, Denu JM, Bäckhed F, Lusis AJ, et al. Interactions between roseburia intestinalis and diet modulate atherogenesis in a murine model. Nat Microbiol. 2018;3(12):1461–1471. doi:10.1038/s41564-018-0272-x. 30397344 PMC6280189

[108] Nagpal J, Cryan JF. Microbiota-brain interactions: moving toward mechanisms in model organisms. Neuron. 2021;109(24):3930–3953. doi:10.1016/j.neuron.2021.09.036. 34653349

[109] O’Donnell MP, Fox BW, Chao PH, Schroeder FC, Sengupta P. A neurotransmitter produced by gut bacteria modulates host sensory behaviour. Nature. 2020;583(7816):415–420. doi:10.1038/s41586-020-2395-5. 32555456 PMC7853625

[110] Chen L, Zhernakova DV, Kurilshikov A, Andreu-Sanchez S, Wang D, Augustijn HE, Vich Vila A, Lifelines Cohort S, Weersma RK, Medema MH, et al. Influence of the microbiome, diet and genetics on inter-individual variation in the human plasma metabolome. Nat Med. 2022;28(11):2333–2343. doi:10.1038/s41591-022-02014-8. 36216932 PMC9671809

[111] Jin WB, Li TT, Huo D, Qu S, Li XV, Arifuzzaman M, Lima SF, Shi HQ, Wang A, Putzel GG, et al. Genetic manipulation of gut microbes enables single-gene interrogation in a complex microbiome. Cell. 2022;185(3):547–562.e22. doi:10.1016/j.cell.2021.12.035. 35051369 PMC8919858

[112] Morreale FE, Kleine S, Leodolter J, Junker S, Hoi DM, Ovchinnikov S, Okun A, Kley J, Kurzbauer R, Junk L, et al. BacPROTACs mediate targeted protein degradation in bacteria. Cell. 2022;185(13):2338–2353.e18. doi:10.1016/j.cell.2022.05.009. 35662409 PMC9240326

[113] Han W, Tellez LA, Perkins MH, Perez IO, Qu T, Ferreira J, Ferreira TL, Quinn D, Liu ZW, Gao XB, et al. A neural circuit for gut-induced reward. Cell. 2018;175(3):665–678 e23. doi:10.1016/j.cell.2018.08.049. 30245012 PMC6195474

